# Early pro-inflammatory cytokine elevations in the DBA/2J mouse model of glaucoma

**DOI:** 10.1186/s12974-015-0399-0

**Published:** 2015-09-17

**Authors:** Gina N. Wilson, Denise M. Inman, Christine M. Denger-Crish, Matthew A. Smith, Samuel D. Crish

**Affiliations:** Department of Pharmaceutical Sciences, Northeast Ohio Medical University, 4209 State Route 44, Rootstown, OH 44272 USA; Biomedical Sciences Graduate Program, Kent State University, 800 E. Summit Street, Kent, OH 44240 USA; Integrated Pharmaceutical Medicine Graduate Program, Northeast Ohio Medical University, 4209 State Route 44, Rootstown, OH 44272 USA

**Keywords:** Cytokines, Glaucoma, Inflammation, Intraocular pressure, Aging, Neurodegeneration

## Abstract

**Background:**

Neuroinflammation—astrogliosis, microglial activation, and changes in cytokine signaling—is a prominent feature of neurodegenerative disorders. Glaucoma is a group of chronic neurodegenerative conditions that make up the leading cause of irreversible blindness worldwide. Neuroinflammation has been postulated to play a significant role in the pathogenesis and progression of glaucomatous neurodegeneration. Though much is known regarding inflammation in the eye in glaucoma, little is known about cytokine activity outside of the retina where pathologies develop early.

**Methods:**

We traced the primary visual projection from the eye to the superior colliculus (SC) in DBA/2J and DBA/2J.*Gpnmb*^*+*^ (control) mice using the anterograde tracer cholera toxin-B (CTB) to assay axonal transport deficits. Forty-eight hours later, visual structures were microdissected from fresh tissue based on transport outcome. Using magnetic bead multiplexing assays, we measured levels of 20 cytokines in the retina, proximal and distal optic nerves, CTB-positive and negative SC subdivisions, cerebellum, and serum at different ages representing different stages of pathology.

**Results:**

Pro- and anti-inflammatory cytokine levels in mice often changed in the same direction based on strain, age, and tissue. Significant elevations in retinal pro-inflammatory cytokines were observed in young DBA/2J mice compared to controls, followed by an age-dependent decrease in the DBA/2J mice. Proximal optic nerve of young DBA/2J mice showed a 50 % or greater decrease in levels of certain cytokines compared to older DBA/2J cohorts and controls, while both proximal and distal optic nerve of DBA/2Js showed elevations in IL-1β at all ages compared to controls. Pro-inflammatory cytokine IL-6 levels varied in accordance with transport outcome in the SC: IL-6 was elevated 44–80 % in glaucomatous DBA/2J collicular regions deficient in anterograde transport from retinal ganglion cells (RGCs) compared to areas with intact transport.

**Conclusion:**

Dysregulation of cytokine signaling in the RGC projection of DBA/2J mice was evident early in distal retinal targets, well before intraocular pressure elevation or axonal degeneration begins.

## Background

Defects in axonal transport have been reported among the earliest pathologies in many neurodegenerative disorders [1, 2], including glaucoma [3–6]. Anterograde transport deficits have been found to precede retrograde deficits and overt structural degeneration of retinal ganglion cell (RGC) axons [7], indicating that an intact and semi-functional axon persists after initial onset of pathology. Furthermore, astrogliosis occurs in the superior colliculus (SC) after transport deficits but before axon loss [8]. Given the early appearances of axon transport deficits and neuroinflammation, obvious questions concern the relationship between these two processes. While much has been reported on inflammation in the retina and optic nerve head, details of immune dysfunction such as changes in cytokine levels have not been examined further along the retinal projection—an area where we first see transport deficits and degeneration [4, 9, 10].

Glaucomatous neurodegeneration is predicted to afflict nearly 80 million people worldwide by the year 2020 [11]. Age and elevated intraocular pressure (IOP) are major risk factors for glaucoma, with IOP currently comprising the only target of FDA-approved drug treatments. However, what actually blinds in the disease is the dysfunction and degeneration of RGCs [12, 13]. Also, given the common incidence of elevated IOP without glaucomatous vision loss as well as normal tension glaucoma, it is clear that many other factors play a role in the development and progression of this degenerative disease [14–16]. Abnormal activation of the immune system has been shown to produce glaucomatous pathology in the absence of elevated IOP [14]. Bosco and colleagues have shown microglial activation within young DBA/2J mouse retina, pre-laminar optic nerve, and nerve head [17, 18], which suggests a role for immunomodulatory molecules such as cytokines early in pathology in moderately elevated IOP contexts. At the other extreme, models of high IOP and nerve crush have also demonstrated some reliance on immunomodulation for either protection [19] or propagation of damage [20].

Cytokine activity is thought to play a substantial part in anterior chamber changes that can result in elevated IOP [21, 22]. Interestingly, increased IL-18 within anterior chamber structures has been evident even in young DBA/2J mice before they exhibit elevated IOP [21]. Additionally, increases in IL-6-type cytokines and members of the JAK-STAT (Janus kinase signal transducer and activator of transcription) signaling pathway were shown in early pressure-induced optic nerve head (ONH) injury [23]. Microglial release of IL-6 in mixed cultures of RGCs, microglia, and astrocytes in response to hydrostatic pressure illustrates an important link between cytokine levels and risk factors for developing glaucoma [24]. Upregulation of genes associated with immune responses (e.g., *Edn2*) have been localized to the retina and ONH of DBA/2J mice very early on in this disease model, and therapeutic attempts to block these responses have shown promise in ameliorating glaucomatous pathology [25]. Previous characterizations of cytokine levels in the context of glaucoma and related stressors, however, being primarily isolated to portions of the anterior chamber or retina, fail to tell the entire story regarding inflammatory signaling in glaucomatous neurodegeneration.

Observations of microglial status within the proximal portion of the RGC projection, in conjunction with cytokine measurements in the eye, suggest that inflammatory signaling may be an early and ubiquitous dysfunction that appears prior to clinically diagnosable vision loss. Therefore, it would be beneficial to assess cytokine levels throughout the entire RGC projection, especially given the observation of anterograde transport deficits and distal axonopathy in glaucoma [4]. Furthermore, elucidating any relationship between cytokine levels and axonal transport outcome to the SC is important in moving towards a clinically relevant means of preventing, halting, or treating the disease. If changes in cytokine signaling do occur within the RGC projection prior to transport loss and RGC death, such findings could open the door for potential therapies that focus on restoring and maintaining function rather than replacing lost structure or treating risk factors, such as IOP, in lieu of the actual mechanisms that drive vision loss.

## Methods

### Animals

**Fig. 1:**
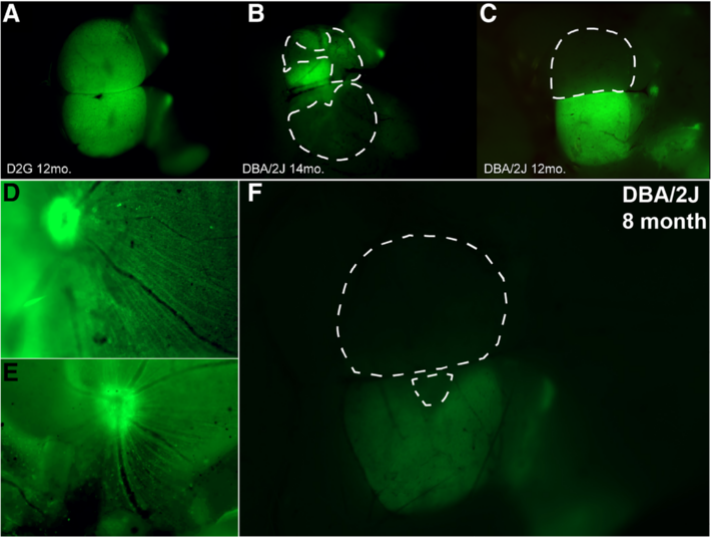
Coverage of cholera toxin-B (CTB) transport in the superior colliculus (SC). **a**–**c** Whole mount brain (cortex removed) of three mice demonstrating varying degrees of anterograde transport to major retinal targets that include lateral geniculate nucleus (LGN), pretectum (PT), and SC. **a** Colliculi taken from a 12-month-old D2G control mouse show intact axonal transport with full CTB coverage in both right and left SC. **b** Colliculi taken from a 14-month-old DBA/2J mouse show compromised transport; left SC has 56 % CTB coverage, right SC has 18 % CTB coverage. **c** Colliculi taken from 12-month-old DBA/2J mouse demonstrate both complete CTB dropout (left SC, 0 % CTB) and complete CTB coverage (right SC, 100 % CTB). *Dotted lines* indicate delineation used for microdissections of CTB-negative (i.e., transport absent) areas. **d, e** Whole mount retinas with successful CTB uptake via intravitreal injection. **f** Whole mount SC with areas of transport loss, corresponding to retinas in **d** and **e**. Retinal images (**d**, **e**) provide evidence that lack of CTB in SC is not indicative of failed intravitreal injection or lack of CTB uptake

Mixed-sex DBA/2J (*n* = 32; 10–12 per age group) and DBA/2J-*Gpnmb*^*+*^ (*n* = 14; 6–8 per group) mice of different ages were used for these studies (refer to Table 1 for specific group nomenclature and group n values). The DBA/2J mouse has two loss of function mutations that produce iris atrophy resulting in age-related elevation of IOP and progressive degeneration of visual structures that mimic human glaucoma [26]. DBA/2J-*Gpnmb*^*+*^ mice (D2G) have the same background as DBA/2J mice; however, they express a functioning wild-type *Gpnmb*^*+*^ allele that prevents them from developing elevated IOP or glaucomatous pathology [27]. All animals were originally obtained from The Jackson Laboratory (Bar Harbor, ME, USA) and were then housed and aged in the Comparative Medicine Unit at Northeast Ohio Medical University. As the DBA/2J model has shown pathological variability in the literature, we based our age groups for comparison on previous work published by ours and other lab groups [4, 7, 8, 28] and included the following: 3–5-month-old mice (D3–5) representing pre-glaucomatous ages, 8–10-month-old mice (D8–10) representing early glaucomatous pathology where anterograde transport deficits and mild axonopathy are evident, and 12–15-month-old mice (D12–15) representing increasing transport deficits and axonopathy characteristic of late glaucomatous pathology [4, 7, 8, 28]. For controls, we used 3–5-month- and 12–15-old D2G mice to represent ages targeted for pre-glaucomatous and late glaucomatous time points (G3–5 and G12–15, respectively). Staging of glaucomatous mice was based on previous work from our lab as well as others (refer to [4, 7, 28]) and was based on the loss of anterograde and retrograde transport and eventual RGC loss. We examined all retinas before tissue preparation to determine that our tracer injections succeeded and we did not see large-scale loss of RGCs or diminished uptake of CTB (Fig. 1). All mice were maintained in the same housing unit under a 12-h light/dark cycle with standard rodent chow available ad libitum. All experimental procedures were approved by the Northeast Ohio Medical University Institutional Animal Care and Use Committee.

### Intravitreal injection of CTB

Animals were anesthetized with 2.5 % isoflurane and placed prone in a stereotaxic device (Stoelting, Wood Dale, IL). Cholera toxin B-subunit conjugated to Alexa Fluor 488 (CTB) was injected into both eyes intravitreally (1.5 μl of 0.1 % CTB in sterile phosphate-buffered saline (PBS) per eye; Life Technologies: Grand Island, NY) using a 33-gauge needle attached to a 25 μl Hamilton syringe. Forty-eight hours later, animals were sacrificed by decapitation under 2.5 % isoflurane anesthesia.

### Microdissection and tissue collection

Retina, ONs, SC, and cerebellum (as a non-glaucomatous control tissue) were collected and immediately frozen on dry ice. Whole blood samples were collected in tubes containing 500 mM EDTA on wet ice. ONs were bisected for analysis of the proximal portion (pON) and distal portion (dON) separately. Whole-mount SC were imaged using a Zeiss AxioZoom V16 epifluorescent microscope equipped with a digital high-resolution camera (AxioCam MRm Rev.3; Zeiss, Jena, Germany). Under ×16.2 magnification, SC were microdissected into “transport intact” (i.e., “CTB-positive”) and “transport absent” (i.e., “CTB-negative”) samples which were analyzed separately in order to parse the relationship between transport outcome and protein levels (Fig. 1). Retinas were flattened and examined to determine success of the tracer injection.

Whole blood samples were centrifuged at 2000×*g* for 10 min at 4 °C. Supernatants were collected and centrifuged for 10 min at 10,000×*g* and 4 °C. Final supernatant was used for serum analyses as a control for global changes in inflammatory markers. Samples were stored at −80 °C until use.

### Tissue processing and multiplex procedure

Tissue samples were rinsed with cold PBS to remove residual blood. Samples were homogenized via sonication (10 % amplitude for two 2-s pulses) approximately 200 μl of 0.1 % Igepal Ca-630 detergent dissolved in PBS with protease inhibitor cocktail (1× Halt Cocktail; Thermo Fisher, Waltham, MA, USA) and centrifuged at 1000×*g* for 10 min after which supernatants were collected for further analyses. We obtained 150–230 μg of protein from each half of the optic nerve and greater than 500 μg protein each from the retina and SC samples. These amounts were sufficient to perform our bead-based multiplex assays, so samples were not pooled.

Multiplex assays were conducted in flat-bottom 96-well plates according to manufacturer’s instructions using reagents provided with the kit (Invitrogen Mouse 20-plex Cytokine Panel, Cat# LMC0006M; Life Technologies: Grand Island NY, USA). In brief, 50 μl of antibody-coupled bead solution was pipetted into each well. Samples were diluted 1:4 in assay diluent to a total volume of 100 μl. One hundred microliters of reconstituted standard was serially diluted (1:3) and added to appropriate wells. Fifty microliters of Igepal buffer diluted in 50 μl assay buffer was used as a blank. Finally, 50 μl of incubation buffer was added to all wells. Plates were kept light-protected during an overnight incubation (4 °C, 16–18 h) on a titer plate shaker at 600 rpm.

Following sample incubation, plates were washed with a mild detergent solution using a magnetic 96-well separator three times (1 min per wash). One hundred microliters of horseradish peroxidase-conjugated detection antibody was added to each well and incubated at RT for 1 h (shaking at 600 rpm). Plates were washed three times and 100 μl of streptavidin-rpe was added to each well. After the 30-min streptavidin-rpe incubation, plates were again washed three times followed by a final addition of 125 μl wash solution to all wells. After 3 min of vigorous shaking, plates were read on a Luminex Magpix unit (Life Technologies, Grand Island, NY, USA) and initial analyses were performed by Xponent software. Results were exported into Microsoft Excel for further processing. Sample size was set to 50 μl, and minimum count was set to 100 events/bead region.

### Bicinchoninic acid assays for total protein concentration

An aliquot of each tissue homogenate was diluted 1:10 in phosphate buffer, and total protein content was assessed in tissue samples using bicinchoninic acid (BCA) assay, following manufacturer’s instructions (Cat# 23225, Thermo Fisher, Waltham, MA, USA). Cytokine levels were all normalized to total protein concentration by dividing cytokine concentration (pg/ml) by total protein (μg/ml) within each tissue extract. Samples were not pooled.

### FGF-2 immunofluorescence and microscopy

Whole mount retina and retinal cross sections from representative animals in each age group, traced with CTB, were assayed for fibroblast growth factor (FGF)-2 localization. Since FGF-2 is present at higher levels than most other cytokines, and it showed specific differences between groups in multiplex analyses, it was chosen for immunostaining to determine if multiplex differences could be supported by histology.

Tissue was collected from animals given a lethal dose of sodium pentobarbital (120 mg/kg, ip.) and transcardially perfused with 4 % paraformaldehyde. The eyes were enucleated and postfixed for 2 h, and then the tissue was placed in a 20 % sucrose cryoprotectant solution (%*w*/*v* in PBS) prior to dissection. Retina designated for cross-sectioning were embedded in gelatin, and 10-μm sections were taken on a cryostat.

Whole retina were assayed as free-floating sections while cross sections were assayed on slide. The tissues were blocked with a 5 % normal donkey serum and 1 % Triton-X 100 in PBS for 2 h. Then, the tissues were incubated for 48 h at 4 °C in the following primary antibody cocktail (diluted in 3 % serum, 1 % Triton in PBS): rabbit polyclonal anti-FGF-2 (1:200, Santa Cruz, SC-7911; Dallas, TX) and mouse monoclonal anti-GFAP (1:500, Millipore, MAB360; Billerica, MA). Following three 10-min washes with PBS, secondary antibody cocktail was added for 2 h at room temperature, which contained donkey anti-rabbit Alexafluor 594 (1:250, Jackson Laboratories, 711-586-152; West Grove, PA) and donkey anti-mouse Alexafluor 647 (1:250, Invitrogen, A-31571; Grand Island, NY). The tissue was then rinsed three times with PBS and mounted and coverslipped with Fluoromount-G (Southern Biotech, Birmingham, AL).

Retinas were photographed with a Zeiss Axio Imager M2 epifluorescent microscope equipped with a digital high-resolution camera (AxioCam MRm Rev.3; Zeiss, Jena Germany) and a computer-guided motorized X-Y stage. An apotome module (Zeiss Apotome.2) was used to collect z-stack of images using structured illumination.

### Statistical analyses

Data from XPonent and Excel were imported into IBM SPSS for Windows 22.0 (IBM Corp., Armonk, NY, USA) for statistical analysis. Multicolinearity between variables was observed, necessitating the use of univariate analyses. In order to compare relationships between the different cytokines and minimize the skewed distributions, a log_10_(*x* + 1) transformation was used to normalize data. Results are reported as mean log (cytokine concentration + 1) ± SEM. The log_10_(*x* + 1) was chosen to minimize negative skewedness that would arise due to values between 0 and 1 [29].

Except in the case of distal optic nerve, few differences between cytokine levels in young and old control D2G mice were observed by two-tailed, independent samples *t* tests. Therefore, to simplify analysis and result presentation, data from these mice were combined into one D2G control group. In cases where age-dependent effects were indicated for young and old D2G controls, these data are either shown graphically or noted within the results.

One-way analyses of variance (ANOVAs) were used to test for differences in cytokine levels among groups of mice categorized by age and strain [D2G, D3–5, D8–10, D12–15]. Tukey’s honestly significant difference (HSD) post hoc measures were used to identify specific differences between age and strain groups. Separate analyses were performed for each brain structure (retina, pON, dON, SC, cerebellum) and serum. Due to multiple differences observed in dON between G3–5 and G12–15 mice via *t* tests, subsequent post hoc comparisons separated G3–5 and G12–15 groups for analysis. Cytokines included in analyses were FGF-2, interleukin-1β (IL-1β), interleukin-1α (IL-1α), interleukin-10 (IL-10), interleukin-13 (IL-13), interleukin-6 (IL-6), interleukin-12 (IL-12), interleukin (IL-17), macrophage inflammatory protein-1α (MIP-1α), interleukin-5 (IL-5), vascular endothelial growth factor (VEGF), tumor necrosis factor-α (TNF-α), interleukin-2 (IL-2), interleukin-4 (IL-4), monokine induced by interferon-γ (MIG), monocyte chemoattractant protein-1 (MCP-1), keratinocyte chemoattractant (KC), and interferon-γ (IFN-γ).

For subdivisions of SC based on transport outcome, ANOVAs were run comparing “CTB-positive” and “CTB-negative” tissue for the relevant age/strain groups (as anticipated, D3-5 and D2G animals did not have CTB-negative variables due to intact transport). For purposes of clarity, graphical representations usually include only those cytokines that showed significant differences between groups. GM-CSF and IP-10 were eliminated from statistical analyses due to inconsistent values within and between groups and low number of structures registering readable levels of these cytokines. Pearson product-moment correlation coefficients were computed to assess the relationship between individual cytokine levels in the retina and SC for each strain and age. Alpha for all tests was set at 0.05.

## Results

### Early elevations in cytokine levels within the DBA/2J retina

**Fig. 2:**
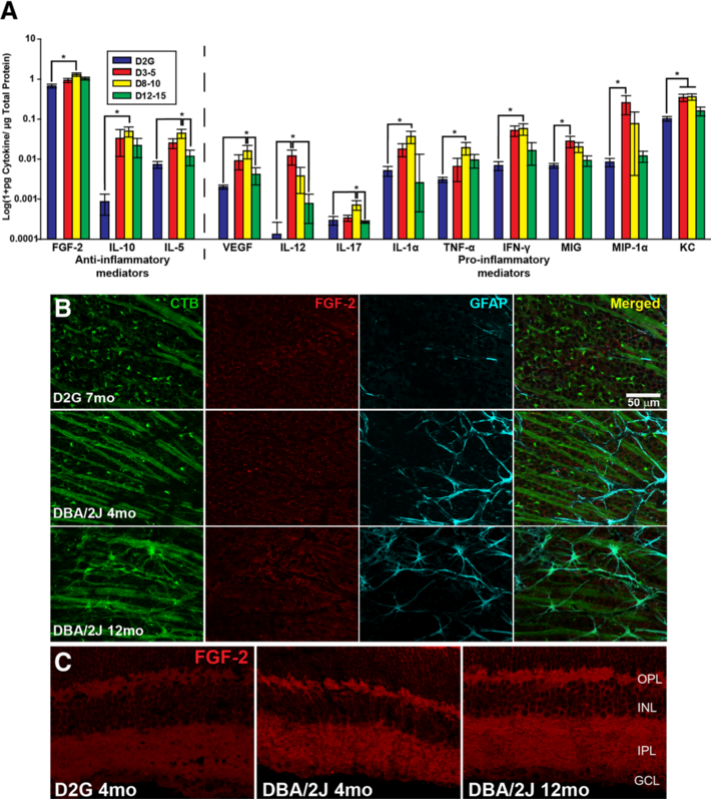
Cytokine protein levels in retina. Anti-inflammatory mediators (FGF-2, IL-10, and IL-5) were elevated in 8–10-month DBA/2J mice and often demonstrated an age-dependent decrease (**a**). A number of pro-inflammatory mediators, such as TNF-α, were also elevated in D8–10 mice; others, such as IL-12 and MIP-1α, were elevated in D3–5, often demonstrating an age-dependent decrease. *Error bars* depict standard error of the mean. *Asterisk* indicates that the group has significantly greater levels than bracketed comparison (*p* < 0.05). **b** Photomicrographs depicting CTB (*first column*) and elevated immunostaining for FGF-2 (*second column*) and GFAP (*third column*) in representative DBA/2J retina whole mount compared to a D2G. Merged images for all stains are shown in the *fourth column*. **c** Photomicrographs of elevated FGF-2 immunostaining in retinal cross sections of representative DBA/2J compared to D2G. Scale bar = 50 μm

We found significantly elevated anti-inflammatory FGF-2 in 8–10-month DBA/2J retinas compared to D2G controls (*F*_3, 38_ = 6.937, *p* < 0.001), with a trend towards increased FGF-2 in the D12–15 group (*p* = 0.08). Anti-inflammatory cytokines IL-5 (*F*_3, 39_ = 5.686, *p* = 0.003) and IL-10 (*F*_3, 25_ = 5.526, *p* = 0.006) were also elevated in the retina of D8–10 mice compared to controls. The following pro-inflammatory cytokines were elevated early in D3–5 mouse retina compared to controls: VEGF (*F*_3, 39_ = 3.098, *p* = 0.039), IL-12 (*F*_3, 39_ = 3.982, *p* = 0.015), IL-17 (*F*_3, 39_ = 3.693, *p* = 0.020), IL-1α (*F*_3, 36_ = 2.924, *p* = 0.048), TNF-α (*F*_3, 38_ = 3.051, *p* = 0.041), IFN-γ (*F*_3, 37_ = 3.904, *p* = 0.017), MIG (*F*_3, 39_ = 3.453, *p* = 0.030), MIP-1α (*F*_3, 30_ = 3.255, *p* = 0.032), and KC (*F*_3, 39_ = 6.311, *p* = 0.009). KC also remained elevated in the D8-10 retina (*p* = 0.005) prior to an age-dependent decrease also observed in other cytokines (see Fig. 2). Additionally, the following pro-inflammatory cytokines peaked at 8–10 months of age in DBA/2J retinas, reaching levels that were significantly greater than D2G controls: VEGF (*F*_3, 39_ = 3.098, *p* = 0.032), IL-17 (*F*_3, 39_ = 3.693, *p* = 0.034), IL-1α (*F*_3, 36_ = 2.924, *p* = 0.029), TNF-α (*F*_3, 38_ = 3.051, *p* = 0.029), and IFN-γ (*F*_3, 37_ = 3.904, *p* = 0.033).

No significant differences between old and young D2G retina were shown for the following cytokines: FGF-2 (*t*_(9)_ = −2.173, *p* = 0.058), IL-10 (*t*_(6)_ = 0.389, *p* = 0.711), IL-5 (*t*_(10)_ = −0.166, *p* = 0.871), VEGF (*t*_(10)_ = 0.339, *p* = 0.742), IL-12 (*t*_(10)_ = 1.000, *p* = 0.341), IL-17 (*t*_(10)_ = 1.213, *p* = 0.92), IL-1α (*t*_(10)_ = 0.552, *p* = 0.593), TNF-α (*t*_(10)_ = −0.411, *p* = 0.690), IFN-γ (*t*_(9)_ = −0.333, *p* = 0.747), MIG (*t*_(10)_ = −0.385, *p* = 0.708), MIP-1α (*t*_(10)_ = 1.206, *p* = 0.255), and KC (*t*_(10)_ = −0.957, *p* = 0.361) (data not shown).

### Select pro-inflammatory cytokines are elevated in pON of DBA/2J mice

**Fig. 3:**
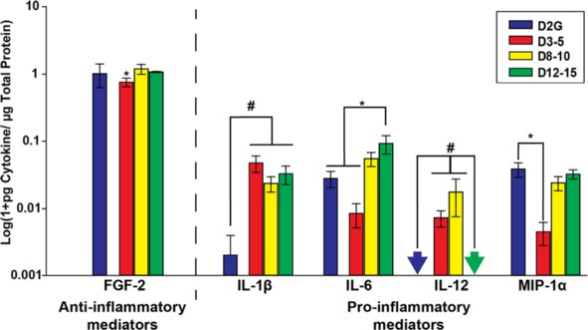
Cytokine protein levels in proximal optic nerve (pON). Anti-inflammatory FGF-2 and pro-inflammatory IL-6 and MIP-1α levels were lower in the pON of young DBA/2J mice than all other groups. Pro-inflammatory IL-12 was only elevated in pON of young and middle-aged DBA/2J mice. *Asterisk* indicates that D3–5 mice have significantly lower cytokine levels than D2G controls and D8–10 and D12–15 pON. *Number sign* indicates significance greater than bracketed comparison (*p* < 0.05). *Arrows* indicate values below limits of detection for the assay. *Error bars* depict standard error of the mean

Unlike the retina, where cytokines often reached peak values at D3–5 or D8–10 age groups, cytokines showed a number of different responses in pON of DBA/2J mice (Fig. 3). The levels of the anti-inflammatory modulator, FGF-2, were significantly lower in pON of D3–5 mice compared to all other groups (*F*_3, 36_ = 6.577, *p* < 0.05).

While the pro-inflammatory cytokine, MIP-1α, was significantly lower in pON of D3–5 mice compared to controls (*F*_3, 36_ = 4.125, *p* = 0.009), others showed elevations in DBA/2J groups compared to controls. Pro-inflammatory IL-6 was elevated in D12–15 mice compared to controls and D3–5 mice (*F*_3, 34_ = 6.041, *p* = 0.014). IL-12, on the other hand, was significantly elevated in D3–5 and D8–10 mice compared to controls and D12–15, which showed little to no IL-12 in pON (*F*_3, 28_ = 3.682, *p* = 0.026). Finally, IL-1β was elevated in all DBA/2J groups compared to controls (*F*_3, 15_ = 5.948, *p* = 0.036). (Data are illustrated in Fig. 3).

No significant differences were seen between old and young D2G pON in the following cytokines: FGF-2 (*t*_(10)_ = −1.921, *p* = 0.084), IL-1β (*t*_(4)_ = 1.633, *p* = 0.178), IL-6 (*t*_(9)_ = 0.003, *p* = 0.998), IL-12 (no IL-12 was detected in D2Gs, so *t* test not computed), MIP-1α (*t*_(10)_ = 0.450, *p* = 0.662). However, MCP-1 (*t*_(6)_ = 4.130, *p* = 0.006) and IL-5 (*t*_(9)_ = 2.871, *p* = 0.018) were significantly reduced in old D2G pON compared to young D2G pON; MCP-1 showed the opposite trend in DBA/2J pON, and IL-5 was not significantly different among DBA/2J pONs across ages. D2G controls were combined since no differences were shown among cytokines that changed significantly for the D2 pON (data not shown).

### Age-dependent effects in the distal ON of D2G controls are absent in DBA/2J mice

**Fig. 4:**
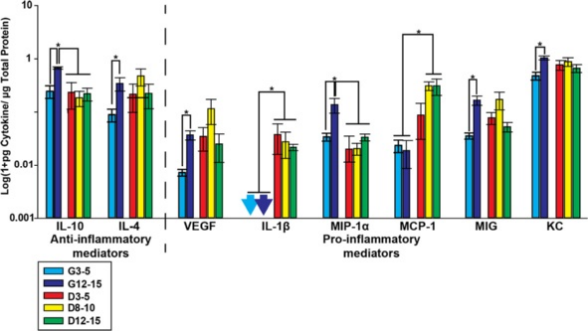
Cytokine protein levels in distal optic nerve. For all cytokines shown, with the exception of IL-1β and MCP-1, there was a significant age-dependent increase in D2G control mice that was not apparent in DBA/2J mice. Pro-inflammatory IL-1β was only detected in DBA/2J mice (*arrows* indicate values below the detection limit of assay). *Asterisk* indicates that the group has significantly higher levels than bracketed comparisons (*p* < 0.05). *Error bars* show the standard error of the mean

Few significant differences were observed in dON among DBA/2J groups (Fig. 4). Pro-inflammatory IL-1β was elevated in dON of all DBA/2J mice compared to that of controls (*F*_3, 26_ = 3.274, *p* = 0.039); notably, this pro-inflammatory cytokine was not detected in D2G mice at all, but was detected in all DBA/2J groups to some degree. Also, pro-inflammatory MCP-1 was significantly elevated in D8–10 and D12–15 dON compared to controls (*F*_3, 23_ = 3.186, *p* = 0.046).

Interestingly, unlike other tissue types, the dON showed a variety of differences between young and old D2G mice. Therefore, analyses used the separated D2G groups as described previously (G3–5 and G12–14), along with D3–5, D8–10, and D12–15. Pro-inflammatory cytokines and growth factors that showed a significant elevation in G12–15 compared to G3–5 mice were as follows: VEGF (*t*_(10)_ = 4.025, *p* = 0.002), MIG (*t*_(9)_ = 3.572, *p* = 0.006), and KC (*t*_(10)_ = 4.698, *p* = 0.001). MIP-1α was significantly elevated in G12–15 compared to G3–5 and all DBA/2J groups (*F*_4, 39_ = 7.060, *p* ≤ 0.009).

Anti-inflammatory cytokines that showed an age-dependent elevation between G3–5 and G12–15 were IL-10 (*t*_(6)_ = 5.831, *p* = 0.001) and IL-4 (*t*_(10)_ = 2.576, *p* = 0.028). Notably, G12–15 mice had significantly increased anti-inflammatory IL-10 levels compared to DBA/2J mice of all ages (*F*_4, 29_ = 5.107, *p* = 0.029). (Data are illustrated in Fig. 4).

### Early increases in pro-inflammatory cytokine levels in the DBA/2J SC

**Fig. 5:**
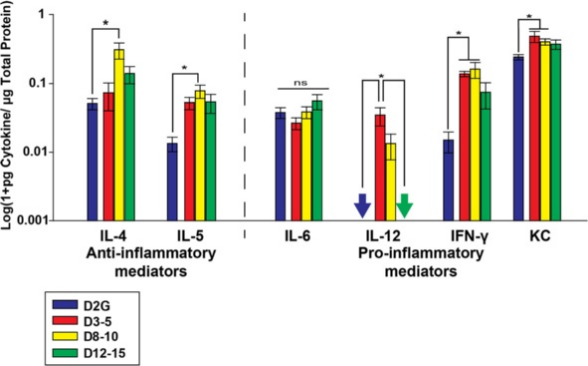
Cytokine protein levels in superior colliculus (SC). Anti-inflammatory IL-4 and IL-5 were elevated in D8–10 mice, while pro-inflammatory IFN-γ and KC were elevated in D3–5 and D8–10 DBA/2J mice compared to controls. Pro-inflammatory IL-12 levels were increased in D3–5 DBA/2J mice compared to controls and 12–15 DBA/2J mice. *Arrows* indicate levels below the detection limit of the assay. *Asterisk* indicates that the group has significantly higher levels than bracketed comparisons (*p* < 0.05). *Error bars* indicate the standard error of the mean

Early increases in cytokine levels were observed in SC of DBA/2J mice (without accounting for transport differences) compared to D2G controls. Anti-inflammatory modulators IL-4 (*F*_3, 40_ = 5.605, *p* = 0.003) and IL-5 (*F*_3, 41_ = 3.713, *p* = 0.019) were both significantly elevated in D8–10 mice compared to controls. Pro-inflammatory modulators, IFN-γ (*F*_3, 31_ = 4.352, *p* = 0.012) and KC (*F*_3, 42_ = 6.035, *p* = 0.002), were significantly increased in both D3–5 mice and D8–10 mice compared to D2G. Pro-inflammatory IL-12 was detected at extremely low levels in controls and old DBA/2J SC, but in respect to these values was increased in D8–10 mice and levels were significantly higher in D3–5 mice (*F*_3, 37_ = 5.021, *p* = 0.005). (Data are illustrated in Fig. 5).

There were no significant differences between old and young D2G SC for the majority of cytokines, including those illustrated (IL-4, IL-5, IL-6 IFN-γ, MIG, IL-12, and KC). However, pro-inflammatory IL-17 was significantly reduced in old D2G SC compared to young D2G cohorts (*t*_(10)_ = 3.820, *p* = 0.003; data not shown).

### IL-6 is the only cytokine that demonstrates a relationship to axonal transport outcome

**Fig. 6:**
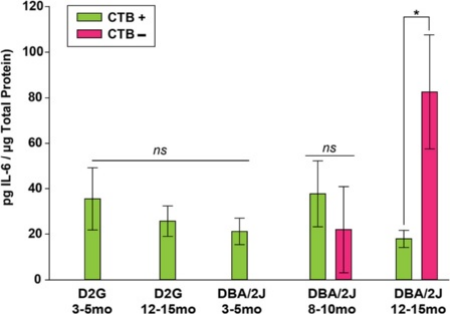
IL-6 levels based on age, strain, and CTB transport to the superior colliculus (SC). Differences in collicular IL-6 levels emerged in 12–15-month-old mice based on transport outcome, with transport absent regions (CTB−) demonstrating significantly greater IL-6 than transport intact (CTB+) regions (*asterisk* indicates *p* < 0.05). There is no anticipated loss of transport in controls and D3–5-month-old groups. *Error bars* show standard error of the mean

Most cytokines did not differ based on anterograde transport outcome of CTB; however, IL-6 was an exception. IL-6 levels were significantly increased in transport-absent SC compared to transport-intact SC (*F*_5, 31_ = 5.543, *p* = 0.001; Fig. 6). This finding is of particular interest because elevation of IL-6 was not apparent when analyzing the SC data before segregation of samples based on transport outcome.

### Cerebellum

No significant differences in cerebellar cytokine levels were observed in DBA/2J and D2G mice across ages (Table 2) indicating that differences in cytokine levels are not generalizable to the nervous system in these mice.

**Table 1:** Summary of group nomenclature and *N* values

Strain	Age	Group nomenclature	Mice per group (*N*)	Projections analyzed (*n*)
D2G	3–5 months	D2G^a^	4	8
D2G	12–15 months		4	6
DBA/2J	3–5 months	D3–5	5	10
DBA/2J	8–10 months	D8–10	6	12
DBA/2J	12–15 months	D12–15	5	10

^a^Young and old D2G mice were combined into a single control group in order to simplify results when possible; independent samples *t* tests indicated that young and old D2G mice showed few differences in their cytokines levels. When detected, differences were described within the results section using G3–5 and G12–15 (referring to young and old D2G mice, respectively)

**Table 2:** Cerebellar cytokine levels across age and strain

	G3–5		G12–15		D3–5		D8–10		D12–15	
	Mean (pg/mg protein)	±SEM	Mean (pg/mg protein)	±SEM	Mean (pg/mg protein)	±SEM	Mean (pg/mg protein)	±SEM	Mean (pg/mg protein)	±SEM
FGF-2	574.40	20.60	562.70	30.40	452.60	12.30	473.30	122.50	531.30	57.20
IFN-γ	2.20	0.90	2.00	1.00	1.80	0.90	6.30	2.90	2.20	1.80
IL-1α	2.50	0.30	1.20	0.30	1.80	0.90	4.40	1.80	2.90	1.60
IL-1β	2.60	0.80	16.80	12.50	7.20	6.20	7.10	3.90	9.60	5.20
IL-2	2.30	0.60	2.50	0.30	1.10	0.60	4.40	2.90	3.10	1.70
IL-4	6.77	5.90	27.50	0.10	21.50	14.00	3.00	0.00	23.80	20.90
IL-5	1.66	0.68	1.80	0.56	3.60	0.91	4.56	2.10	3.01	1.17
IL-6	9.80	1.80	8.70	0.30	5.10	0.80	10.60	4.50	8.70	3.40
IL-10	1.60	0.20	14.60	3.80	8.20	6.00	30.10	27.90	26.80	23.70
IL-13	3.10	3.10	7.90	7.90	2.60	0.90	12.60	7.30	9.87	5.00
IL-12	–	–	–	–	3.30	1.90	0.90	0.90	–	–
IL-17	0.20	0.03	0.04	0.02	0.04	0.01	0.30	0.10	0.20	0.09
KC	215.55	32.76	99.25	67.50	133.07	63.85	268.06	66.14	199.67	60.04
MCP-1	N/A	N/A	–	–	–	–	1.90	1.90	1.35	0.69
MIG	86.96	19.48	10.40	2.40	11.73	2.72	12.98	4.86	12.09	4.64
MIP-1α	10.10	0.30	7.80	0.10	9.18	0.60	14.43	5.20	10.30	2.95
TNF-α	0.72	0.72	1.20	0.46	11.70	6.35	1.48	1.48	1.72	1.72

En dashes indicate that all values were below the limit of detection; for such cases, a zero was recorded. N/A indicates that the majority of samples had no reading or an error reading, which is distinguished from a reading that is simply below the limit of detection

### Serum

There were no differences between DBA/2J and D2G serum levels of cytokines, indicating that changes observed in visual system tissues were not globally present. Cytokine measurements in serum showed much higher inter-sample variability than cytokine levels in the other tissue analyzed in this study.

### Relationship between retina and SC cytokine levels

**Fig. 7:**
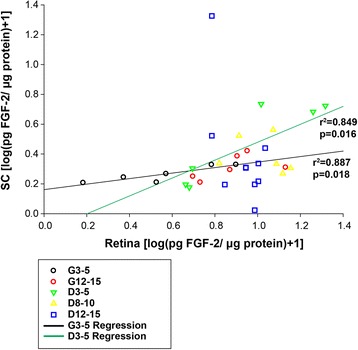
Scatterplot showing relationship between retinal and collicular FGF-2 levels. Symbols represent individual retina versus the corresponding superior colliculus. Significant positive correlations were indicated between retinal and collicular FGF-2 levels for both young D2G (3–5 months) and young DBA/2J (3–5 months) mice (Pearson’s *r* ^2^). *Regression lines* have been drawn for each of these groups to illustrate these relationships. No significant relationships were shown for other groups, including aged D2G mice. *p* values are indicated on the figure

Given the frequent disconnect between pathology in the retina versus targets in the brain, we sought to identify any relationships in cytokine levels between these two structures. Few cytokines showed a strong and consistent relationship between retinal and collicular levels (*p* > 0.05). However, FGF-2 levels in the retina and SC were significantly correlated in our youngest D2G and DBA/2J mice [Fig. 7, G3–5 (*r*^2^ = 0.887, *p* = 0.018); D3–5 (*r*^2^ = 0.849, *p* = 0.016)]. This relationship was not reflected in older animals of either strain. This finding suggests that age, independent of pathology, may disrupt the association between retinal and collicular FGF-2 levels in mice. Pathological changes may further challenge this relationship, as we observed elevations in absolute retinal FGF-2 levels in DBA/2J mice compared to controls (see Fig. 2).

**Fig. 8:**
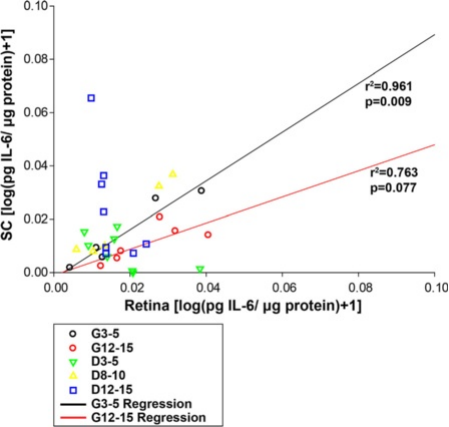
Scatterplot showing relationship between retinal and collicular IL-6. *Symbols* represent individual retina versus the corresponding superior colliculus. A significant, positive correlation between retinal and collicular IL-6 levels in young D2G mice (Pearson’s *r* ^2^) and a strong trend towards this same relationship was indicated in old D2G mice. *Regression lines* have been drawn for each of these groups to illustrate relationships. No significant relationships were shown for other groups. *p* values are indicated on figure

Additionally, IL-6 retinal and collicular levels in young D2G mice were positively correlated, and there was a trend towards this effect in the oldest D2G mice [G3–5 (*r*^2^ = 0.961, *p* = 0.009); G12–15 (*r*^2^ = 0.736, *p* = 0.08)]. No such relationships between retinal and collicular IL-6 levels were shown in any DBA/2J group (Fig. 8). The absence of this relationship in DBA/2J mice at any age indicates that there may be an early and sustained disruption in the association of retinal and collicular IL-6.

### FGF-2 immunofluorescence

Cross sectioned and whole retina show a clear distinction in FGF-2 levels between D2G and DBA/2J groups, with all DBA/2J groups demonstrating increased FGF-2 immunoreactivity (Fig. 2), which supports multiplex data from the retina. The distinction between DBA/2J groups illustrated by multiplex analyses, however, is not as apparent in histological sections. Inner and outer plexiform layers are most densely stained for FGF-2, with diffuse staining throughout other layers of the retina. Data illustrates that the retinal ganglion cell layer is not the only location of FGF-2, and therefore, the FGF-2 detected by multiplex analyses is mostly due to its concentration in these other layers. This also suggests that glia and other cell types constitute the majority of FGF-2 production in the retina. Müller glia are likely a major producer of FGF-2 as reported by Hageman and colleagues [30] and others (refer to [31, 32]).

## Discussion

Glaucoma is an age-related degenerative disorder in which microglial abnormalities have been described, especially at the optic nerve head and retina [18, 33]. Specifically, microglia are found in activated, ramified clusters within the glaucomatous optic nerve head [33]. However, despite the initial changes to ONH, we have previously reported that pathology, presumably due to these changes, manifests distally in retinal targets such as SC [4]. This disparity between locations of the stressor and manifestation of pathology is commonly seen in neurodegenerative disorders [34]. Despite this pattern of progression, a comprehensive description of cytokine profiles throughout this projection apart from retina and optic nerve head has not been done. Our work provides an age-dependent analysis of cytokine levels throughout the entire retinal projection of the glaucomatous DBA/2J model with respect to functional outcome.

### Cytokine protein levels along RGC projection with age and pathology

Early elevations of classically categorized pro-inflammatory cytokines—specifically IFNγ, MIG, KC, TNF-α, and MIP-1α—observed in the retina and SC may signal the progression of pathological changes in the visual projection of DBA/2J mice prior to functional transport loss and RGC death. While the majority of changes we observed were in pro-inflammatory mediators, our results suggest that anti-inflammatory cytokines may play a compensatory role early in glaucoma prior to transport loss, as evidenced by early elevations in retinal IL-5 and IL-10. Other work has shown that IL-10 plays a role in RGC survival and IL-5 has similar functions [35].

TNF-α is another important pro-inflammatory cytokine that was found to be upregulated in the retina of 8–10-month DBA/2J mice, which is expected given that TNF-α has cytotoxic effects associated with apoptotic signaling [36]. In support of our findings, recent work has shown that TNF-α may actually be protective early in glaucomatous pathology, as TNF-α expression is upregulated immediately following nerve crush and TNF-α deficiency intensifies RGC injury following nerve crush [19]. Previous work from other laboratories has shown increased immunolabeling of TNF-α and its associated death receptor, TNF-R1, in the optic nerve head and retina of glaucomatous human donor eyes [37, 38]. While TNF-α was upregulated primarily in glia of retina and optic nerve head, the TNF-1R was most prominent in RGC bodies, suggesting that RGCs may be especially susceptible to the effects of TNF-α [37, 38]. Conversely, TNF-α has also been shown to have early neuroprotective effects via actions of the less-prominent TNF-R2. While some RGC death can be observed in the 8–10-month DBA/2J retina, the protective effects associated with TNF-R2 may be why there is a temporal delay between observed TNF-α elevations and the age at which RGC death becomes overwhelmingly significant (13–15 months; refer to [7]).

Additionally, FGF-2 upregulation was shown in the retina of 8–10-month-old DBA/2J mice (with trends towards this elevation in D3–5 and D12–15 groups), suggesting that FGF-2 may be an early signal of cellular stress. Recently, the Noda laboratory described the response of microglia to FGF-2 with experiments that demonstrated that (a) degenerating neurons released FGF-2 and that (b) microglia specifically responded to this FGF-2 release by adopting an amoeboid phenotype and migrating towards FGF-2 at sites of neuronal degeneration [39]. In our studies, FGF-2 levels in retina and SC of young D2G mice were similar, but became dissociated in older D2Gs. This age-dependent pattern of FGF-2 expression in the retina and SC was recapitulated in DBA/2J mice, but their overall FGF-2 levels were an order of magnitude above those of the D2G controls. Therefore, while FGF-2 levels may become deregulated between the retina and SC as a feature of *normal* aging, it is likely that the actual magnitude of concentrations and not the relative values between structures are the important factor in DBA/2J pathology.

Immunostaining in the retina also supported multiplex FGF-2 data overall, showing an increase in the DBA/2J retina compared to D2G controls. It is noteworthy that FGF-2 expression was apparent in several layers, specifically the outer and inner plexiform layers of the retina and not just in ganglion cells; this is relevant since the whole retina was analyzed in the multiplexing experiments. FGF-2 is produced by a variety of cell types including Müller glia, RGCs, and other cell types [30, 31]. Furthermore, FGF-2 stimulates Müller cell proliferation [32]. Wen and colleagues also showed that FGF-2 is upregulated in the retina in response to acute, mechanical injury and suggested that this actually serves as a protective response in the context of photoreceptor degeneration [31]. While chronic, glaucomatous neurodegeneration differs distinctly from this acute type of photoreceptor degeneration, it is still a logical extrapolation that our observed FGF-2 elevations within DBA/2J retina could be an intrinsic signal or attempt to by retinal cells to be “rescued.”

While FGF-2 elevations and other anti-inflammatory changes in the SC did differ significantly between DBA/2J age groups, anti-inflammatory IL-4 and IL-5 were elevated in the D8–10 age group. Additionally, a number of pro-inflammatory cytokines were elevated in young DBA/2J mice compared to D2G mice and older DBA/2J mice. For example, KC was elevated in all D3–5 and D8–10 groups compared to D2G mice, which may drive some of the more prolonged inflammation and advanced pathology within the DBA/2J strain. This idea is supported by data in htau mice, a common neurodegenerative model for Alzheimer’s disease, which show elevated KC and MIG, among other pro-inflammatory mediators, within their cortex when compared to controls [40]. Other pro-inflammatory cytokines elevated in D3–5 SC included IL-12 and IFN-γ, which may also perpetuate early pathology prior to transport loss.

Interestingly, distal portions of the ON in control mice showed age-dependent elevations in cytokines—a finding that was absent in aging DBA/2J mice and contrasted with findings in other regions of the visual projection. Some notable changes in dON of D2G mice with age were in levels of anti-inflammatory cytokines IL-10, and IL-4, suggesting that these may be protective elevations that occur in the distal projection of the ON during normal aging. This was corroborated by the finding that DBA/2J mice lacked this response as they aged.

Notably, IL-1β was significantly elevated in both proximal and distal portions of the optic nerve in DBA/2J mice compared to controls, indicating a possible role for this cytokine in distal axonopathy in glaucoma [4]. IL-1β has been shown to be an upstream activator of JNK via transforming growth factor-β activating kinase-1 (TAK1) [41], and hyperphosphorylation of neurofilaments and motor proteins by JNK may alter the substrate and machinery by which axonal transport occurs to sustain cellular and axonal viability [42].

### Cytokines and axonal transport

Largely, changes in cytokine levels were independent of axonal transport outcome in the SC as well as in the corresponding retina. However, due to the small sample size and variability within the DBA/2J strain, these data should be interpreted cautiously. Interestingly, IL-6 was the only molecule that differed based on transport outcome; as expected, IL-6 levels were highest in the most pathological (i.e., transport-absent) SC at 12–15 months. Given that RGC death in DBA/2J mice occurs most significantly at 18 months of age [28] and D8–10 SC showed no differences in IL-6 based on transport, increased IL-6 in transport-deficient SC of D12–15 mice may signal impending RGC loss. This contrasts with reports in the retina, which has shown that an IL-6 transducer was elevated under conditions of glaucoma-related stress before RGC loss [43].

The function of IL-6 in these regions has been suggested to be involved in the regeneration of injured axons in optic nerve trauma and more acute IOP elevation [44]. The chronic degeneration in DBA/2J mice, however, presents a more complicated scenario. While IL-6 levels were significantly reduced in the proximal optic nerve of DBA/2J mice compared to controls, possibly due to transport deficits, these levels increased with age and were significantly greater in the SC of the oldest DBA/2J groups. Given that we do not see extensive axon injury until then [4, 7, 8, 28], elevation of IL-6 within the D12-15 SC suggests that this signaling may be a neuroprotective mechanism attempting to salvage or replace lost connections [44].

We observed that IL-6 levels were highly correlated between the retina and SC of young D2G mice, and there was a trend toward this relationship in the old D2G controls. However, in DBA/2J mice, we did not see a relationship between retinal and collicular IL-6*.* As has been suggested previously [44], IL-6 may be elevated specifically in response to axon damage. Given that we have previously reported distal axonopathy in this model, we would expect elevations in IL-6 to occur in the distal targets (e.g., SC) first, therefore uncoupling the relationship between retina and SC.

## Conclusion

Dysregulation of cytokine signaling throughout the RGC projection of DBA/2J mice is evident early in pathology. Early elevations of classic pro-inflammatory cytokines in the retina—specifically IFNγ, MIG, KC, TNF-α, and MIP-1α as well as consistent elevations in IL-1β throughout the ON may signal the progression of pathological changes in the visual projection of DBA/2J mice prior to functional transport loss and RGC death. Additionally, differences in cytokine levels, either age-related or strain-related, are compartmentalized throughout the RGC projection. Overall, DBA/2J mice show many age-dependent changes in pro-inflammatory cytokines and some alterations in anti-inflammatory cytokines that are not observed in control mice within the retina and proximal ON and SC.

Our data suggest that changes in cytokine and growth factor levels may occur prior to functional transport loss and are consistent with previous work showing inflammatory changes and immune-mediated vasoconstriction early in glaucomatous pathology [25]. Such alterations in immunomodulatory molecules may be among the subtle changes that occur prior to overt cytoskeletal damage, loss of axonal transport, and cell death. However, IL-6 levels were elevated in accordance with axonal transport loss in our oldest DBA/2J mice, indicating that IL-6 may be a reliable marker of late pathological states [44]. Previous work by Luterman and colleagues [45] showed a similar pattern in patients with late/terminal Alzheimer’s disease exhibiting greater amounts of IL-6 mRNA in entorhinal cortex and temporal gyrus compared to controls. This group also demonstrated that levels of IL-6 were positively correlated with neurofibrillary tangles [45]. Thus, IL-6 elevations in the SC may be closely coupled to structural demise and axonal loss, as previous work from our lab has shown that both retrograde transport deficits and structural loss are characteristic of the 12–15-month DBA/2J group [7]. However, the current study did not directly assess this relationship.

Given that our findings indicate a complex role for cytokine signaling throughout the RGC projection, it would be interesting to parse out immune cell phenotypes responsible for cytokine release versus resting, phagocytotic, primed, or senescent phenotypes throughout the projection as a function of strain and age. Additionally, further investigation into the interplay between the immune system and axonal transport disruption via downstream, kinase-mediated cytoskeletal, or motor protein modifications could also provide important insight into the progression and potential therapeutic targets of glaucoma and other related neurodegenerations.
