# Erratum To: Early pro-inflammatory cytokine elevations in the DBA/2J mouse model of glaucoma

**DOI:** 10.1186/s12974-015-0410-9

**Published:** 2015-10-26

**Authors:** Gina N. Wilson, Denise M. Inman, Christine M. Dengler-Crish, Matthew A. Smith, Samuel D. Crish

**Affiliations:** Department of Pharmaceutical Sciences, Northeast Ohio Medical University, 4209 State Route 44, Rootstown, 44272 OH USA; Biomedical Sciences Graduate Program, Kent State University, 800 E. Summit Street, Kent, 44240 OH USA; Integrated Pharmaceutical Medicine Graduate Program, Northeast Ohio Medical University,, 4209 State Route 44, Rootstown, 44272 OH USA

Following the publication of our article [1] we noticed that the author name was included incorrectly. Author name Christine M Denger Crish in the original article should read Christine M Dengler Crish. The original version of the article has now been corrected to reflect this.
